# The Role of Non-Coding RNAs in Neurodevelopmental Disorders

**DOI:** 10.3389/fgene.2019.01033

**Published:** 2019-11-20

**Authors:** Shuang-Feng Zhang, Jun Gao, Chang-Mei Liu

**Affiliations:** ^1^State Key Laboratory of Stem Cell and Reproductive Biology, Institute of Zoology, Chinese Academy of Sciences, Beijing, China; ^2^Savaid Medical School, University of Chinese Academy of Sciences, Beijing, China; ^3^School of Life Sciences, University of Science and Technology of China, Hefei, China; ^4^Department of Neurosurgery, Peking Union Medical College Hospital, Chinese Academy of Medicine Sciences & Peking Union Medical College, Beijing, China; ^5^Institute for Stem Cell and Regeneration, Chinese Academy of Sciences, Beijing, China

**Keywords:** non-coding RNA, neurodevelopmental disorder, miRNA, piRNA, snoRNA, lncRNA

## Abstract

Non-coding RNAs, a group of ribonucleic acids that are ubiquitous in the body and do not encode proteins, emerge as important regulatory factors in almost all biological processes in the brain. Extensive studies have suggested the involvement of non-coding RNAs in brain development and neurodevelopmental disorders, and dysregulation of non-coding RNAs is associated with abnormal brain development and the etiology of neurodevelopmental disorders. Here we provide an overview of the roles and working mechanisms of non-coding RNAs, and discuss potential clinical applications of non-coding RNAs as diagnostic and prognostic markers and as therapeutic targets in neurodevelopmental disorders.

## Introduction

Non-coding RNAs are RNA molecules that are not translated into proteins. Recent advances in genomic sequencing technologies and functional assays enable a more in-depth understanding of their characteristics (Mattick, 2011; Djebali et al., 2012; Obiols-Guardia and Guil, 2017). The transcription process of non-coding RNAs is precisely orchestrated in time and space (Okazaki Y., et al., 2002; Djebali et al., 2012). Different developmental stages or tissue types have distinct transcriptional landscapes (Carninci et al., 2005; Kapranov et al., 2007). The central nervous system is a sophisticated and precise system which is responsible for guiding our daily activities such as sports, learning, emotion and language. Rapidly growing evidence indicated that non-coding RNAs play indispensable roles in brain development, function, and the etiology of neurodevelopmental diseases.

Here, we review the diversity and biogenesis processes of non-coding RNAs, and summarize their versatile roles in neurodevelopmental disorders. We also discuss potential clinical applications of non-coding RNAs as diagnostic and prognostic markers and as therapeutic targets in neurodevelopmental disorders.

## Characteristics of Non-Coding RNAs

Abundant and functionally important types of non-coding RNAs include ribosomal RNAs (rRNAs) and transfer RNAs (tRNAs), as well as regulatory non-coding RNAs which mainly consist of microRNA (miRNA), PIWI-interacting RNA (piRNA), small nucleolar RNA (snoRNA), small interfering RNAs (siRNAs), long non-coding RNA (lncRNA), and Circular RNAs (CircRNAs). Non-coding RNAs play a critical role in epigenetics regulation of gene expression in addition to their roles at the transcriptional and post-transcriptional level (David and Bartel1, 2004; Alexander et al., 2010).

**Figure 1:**
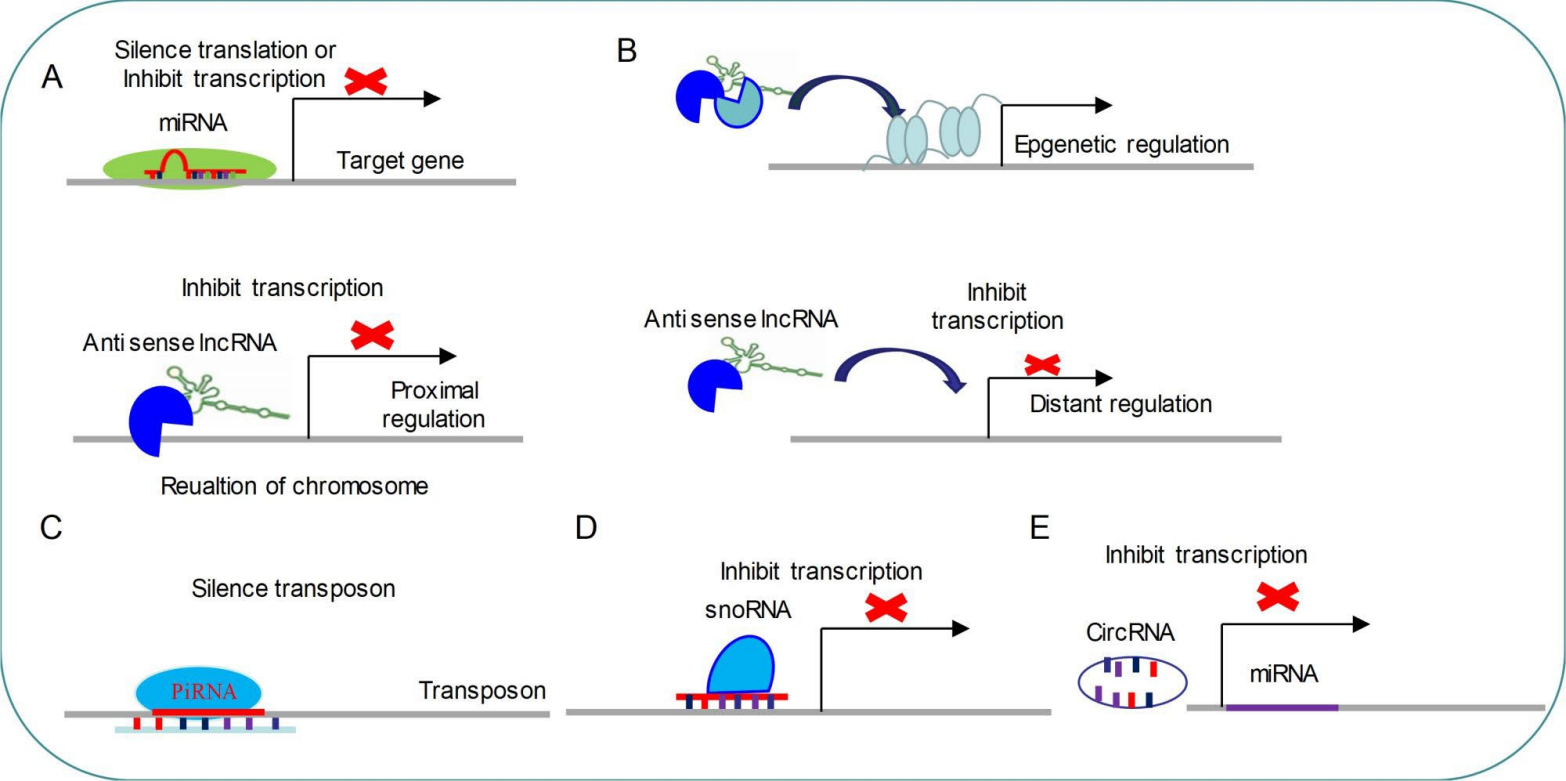
Common regulatory mechanism of non-coding RNAs **(A)** miRNAs modulate target genes by silencing mRNA translation or inhibiting mRNA transcription. **(B)** LncRNAs regulate gene transcription by three distinct ways. **(C)** PiRNAs target transposon for silencing its expression. **(D)** SnoRNAs inhibit transcription by targeting specific loci. **(E)** CircRNAs are able to repress miRNA expressions.

miRNAs are small single-stranded molecules (20–24 nt) that have seed sequences complementary to sequences on target mRNAs transcripts through the 3′UTR, leading to silencing of the target gene. The miRNA gene is transcribed by RNA polymerases II and III to generate a primary microRNA precursor molecule (pri-miRNA). The pri-miRNA then undergoes nuclear cleavage by Drosha/DGCR8 to form a precursor microRNA (pre-miRNA). The pre-miRNA is transported from the nucleus into the cytoplasm by Exportin 5, and then processed by Dicer/TRBP into a miRNA duplex which is unwound by a helicase. The mature miRNA is incorporated into the RNA-induced silencing complex (Risch et al.) which mediates down-regulation of gene expression by either translational repression or mRNA degradation (David and Bartel1; 2004; Esteller, 2011; Rajman and Schratt, 2017) (**Figure 1**).

piRNAs are small non-coding RNAs (24-31 nt) that can silence transposons and regulate gene expression by directing PIWI proteins of Argonaute family to specific genomic loci (Fabio Mohn and Brennecke, 2015) (**Figure 1**). piRNAs biogenesis is divided into primary and secondary pathways (Zamore et al., 2018). In primary pathway, primary piRNAs are transcribed from genomic loci called piRNA clusters (Brennecke et al., 2007; Li et al., 2013). Primary piRNAs are spliced by endonuclease into tail-to-head phased precursor piRNA that are catalyzed by the mitochondrial protein Zucchini/PLD6 (Ipsaro et al., 2012; Nishimasu et al., 2012; Fabio Mohn and Brennecke, 2015). Each pre-piRNA begins with a 5′ monophosphate, a prerequisite for loading RNA into nearly all Argonaute proteins (Schirle et al., 2014; Wang et al., 2014). Once the PIWI protein captures pre-piRNA, the 3′ terminal is trimmed by a single-stranded RNA exonuclease called Trimmer/PNLDC1 to the appropriate size (Kawaoka et al., 2011; Tang et al., 2016; Ding et al., 2017). Finally, the small RNA methyltransferase Hen1/HENMT1 adds a 2-O-methyl moiety to the 3′ ends of the mature piRNAs (Horwich et al., 2007; Lim et al., 2015). The secondary piRNA biogenesis pathway, also known as the “ping-pong” cycle, is a piRNA-directed piRNA synthesis pathway that produces a piRNA *via* interaction with PIWI proteins (Brennecke et al., 2007; Gunawardane et al., 2007; Wang et al., 2014) (**Figure 1**). So far, functions of piRNA are mainly concentrated in regulation of genomic stability *via* silencing transposon (**Figure 1**).

snoRNAs are 60–300 nt nucleotide long, metabolically stable RNAs, which are usually concentrated in Cajal bodies or nucleoli (Ding et al., 2008). snoRNAs are produced by the transcription of RDR2. Compared with miRNA, snoRNA transcription events occur only in the nucleus (Vaucheret, 2006). Initial, transcripts of snoRNA enter the cytoplasm for processing and modifying and subsequently return to the nucleus. snoRNA can catalyze sequence-specific 2′-O-methylation and pseudouridine acidification of ribosomal RNA (rRNA) by forming protein complexes with splicing function (Kiss-László et al., 1996; Jingwei Ni et al., 1997). A new study has found that snoRNAs are indispensable for processing and stability of lncRNA (Xing and Chen, 2018).

lncRNA are a family of long-chain non-coding RNA that are usually longer than 200 nt, which regulate various developmental and physiological processes (Hu et al., 2016; Zhou et al., 2016; Lekka and Hall, 2018). Almost all lncRNAs transcripts do not contain open reading frames (Wilhelm et al., 2014), which are produced by RNA polymerase II, followed by capping and forming 3′polyadenylate tails (**Figure 1**) (Lekka and Hall, 2018). lncRNAs can interact with other epigenetic regulators to direct histone-modified enzymes or DNA-methylated enzymes to the specific gene loci and modulate gene expression (Tripathi et al., 2010; Gong and Maquat, 2011). For example, BDNF-AS can recruit EZH2 and PRC2 complex to the promoter region of BDNF to down-regulate the expression of BDNF (Modarresi et al.). In addition, lncRNAs might act as enhancers to activate gene transcription (Orom et al., 2010), and target miRNAs to silence their inhibitory functions (Cesana et al., 2011; Lai et al., 2013). lncRNAs can also interact with proteins to modulate gene expression in different levels or direct their appropriate spatial subcellular localization (Chu et al., 2015; Lekka and Hall, 2018).

Circular RNAs (CircRNAs) are recently emerged as a new class of endogenous noncoding RNAs (ncRNAs) which might regulate gene expression (Memczak et al., 2013; You et al., 2015). These RNAs are usually processed into loops after transcription(Piwecka et al., 2017). Large-scale sequencing and analysis results have demonstrated that thousands of circular RNAs are present in mammalian and nematodes (Memczak et al., 2013; Liang Chen 2015). The published data also have indicated that CircRNAs are highly abundant in mammalian brain compared to other analyzed tissues (Memczak et al., 2013). What’s more, the majority of detected mouse CircRNAs are also expressed as CircRNAs in human brain, which suggests that CircRNAs are very conserved between species (Liang Chen 2015). Identically, transcription of circular RNA is orchestrated by developmental stage and tissue specificity(Hansen et al., 2013; Liang Chen 2015; Piwecka et al., 2017), which indicates that CircRNAs might serve as regulatory RNAs, especially in brain (**Figure 1**). Loss of a mammalian circular RNA locus causes miRNA deregulation and affects brain function (Hansen et al., 2013; Piwecka et al., 2017). For example, cerebellar degeneration-related protein 1 transcript (CDR1as) contains more than 70 selectively conserved miRNA target sites. CDR1as strongly suppresses miR-7 activity and results in increased levels of miR-7 targets (Hansen et al., 2013; Piwecka et al., 2017). Functionally, CDR1as and its interaction with miRNAs are important for sensorimotor gating and synaptic transmission (Piwecka et al., 2017). Using high resolution *in situ* hybridization technology, the researchers found that visualized CircRNAs punctate into the dendrites of neurons, and many CircRNAs change their abundance abruptly at a time corresponding to synaptogenesis. Together, these data indicate that CircRNAs play important roles in regulating synaptic function (You et al., 2015).

## Non-Coding RNA in Neurodevelopmental Disorders

Human brain displays the richest repertoire of ncRNA species, and where several different ncRNA molecules are known to be involved in crucial steps for neurodevelopment (Mehler and Mattick, 2006; Taft et al., 2010; Obiols-Guardia and Guil, 2017). Abnormal expression of non-coding RNA has been linked with pathologies of several neurodevelopmental diseases, including Autism spectrum disorders (ASD), Fragile X syndrome (FXS), Down syndrome (DS), Rett syndrome, and Prader-Willi Angelman syndrome (**Table 1**).

**Table 1:** Non-coding RNAs are related to neurodevelopmental disorders.

Neurodevelopmental Disorders	Dysregulated Non-coding RNAs	Functional examples	References
Autism Spectrum Disorders	miR-197-5p, miR-328-3p, miR-424-5p, miR-500a-5p, miR-313-5a. Up: miR-619-5p, miR-365a-3p miR-664a-3p, miR-188, RAY1/ST7, ST7OT1-4, ST7AS1-4, ST7OT1-3, SHANK2-AS, MSNP1AS	MiR-188 serves to fine-tune synaptic plasticity by regulating autism susceptibility genes Nrp-2 expression. Overexpression of SHANK2-AS reduces the complexity of neurites, and inhibits the proliferation of neuronal stem cells and promotes their apoptosis	(Talebizadeh et al., 2008; Tewarit Sarachana 2010; Lee et al., 2012; Kerin et al., 2012; Trindade et al., 2013; van de et al., 2013; Mundalil Vasu et al., 2014; DeWitt et al., 2016; Constantin, 2017; Kichukova et al., 2017; Luo et al., 2018)
Fragile X Syndrome	miR-302, miR-125, miR-132, let-7c, miR-9, miR-100, miR-124, miR-125a, miR-125b, miR-127, miR-128, miR-132, miR-138, miR-143, miR-219, FMR4, FMR5, FMR6	MiR-125 and miR-132 interact with FMRP to regulate the signal transduction of metabolic glutamate receptors (mGluR1) and NMDAR. FMR4, FMR5 and FMR6 are detectable in the majority of patient leukocyte RNA samples.	(Ladd et al., 2007; Khalila 2009; Taft et al., 2010; Esteller, 2011; van de et al., 2013; Lin, 2015)
Down Syndrome	miR-99a, let-7c, miR-125b-2, miR-155, miR-802	DS dementia strongly correlates with overexpression of miR-155 on chromosome 21 with concomitant reduction of multiple CNS-functional targets, including BACH1, CoREST1, Cyclin D1, BCL6, BCL10, BIM, and SAPK4.	(Malinge et al., 2009; Fillat and Altafaj, 2012; Asim et al., 2015; Tili et al., 2018)
Rett Syndrome	miR-184, miR-30a, miR-381, miR-495, miR-130a, miR-132, miR-200a, miR-302c, DQ541777, AK081227, AK087060, BDNF-AS	MiR-184, miR-30a, miR-381, and miR-495 are aberrantly up-regulated in MeCP2 knockout mice. These miRNAs are known for repressing the expression of important modulators of neuronal development, such as Bdnf and Numbl. BDNF is known to be aberrantly diminished in RTT individuals, it can be speculated that the lncRNA BDNF-AS might be an important therapeutic target for treating RTT.	(Nomura et al., 2008; Urdinguio et al., 2010; Wu et al., 2010; Petazzi et al., 2013; Lin et al., 2014; Mo 2015; Katz et al., 2016; Rodrigues et al., 2016; Zhang et al., 2016)
Prader-Willi syndrome & Angelman syndrome	SNRPN, SNRD116, Ube3a-ATS	Mice with deletion of SNRD116 (MBII85) snoRNA clusters demonstrated obvious PWS phenotypes. In AS patients, the maternal Ube3a allele is inactive, the paternal allele is intact but epigenetically silenced through the Ube3a-ATS part of LNCAT at the Ube3a locus.	(Muscatelli et al., 2000; Landers et al., 2005; Skryabin et al., 2007; Sahoo et al., 2008; de Smith et al., 2009; Buiting, 2010; Meng et al., 2012)

### Autism Spectrum Disorders

ASD is a developmental disorder that affects communication and behavior, which is characterized by repetitive patterns of behavior, interests, or activities, problems in social interactions, and psychological problems in children (Balachandar et al., 2016). Children with ASD have co-occurring language problems, intellectual disabilities, and epilepsy at higher rates than the general population. While the exact cause of ASD has remained somewhat of a mystery, dozens of genes have been identified to potentially contribute to disease susceptibility (Edward et al., 1985; Bailey et al., 1995; Risch et al., 1999; Klei et al., 2012; Kang, 2014). For example, polymorphisms in the FMR1 gene have been reported to be associated with autism (Reddy, 2005), however, no consistent association between FMR1 polymorphisms and autism has been demonstrated. Therefore, ASD is probably not caused by one single genetic factor. Recent studies suggest that epigenetic mechanisms, such as non-coding RNAs, may play a major role in the pathogenesis of ASD (Constantin, 2017).

**Figure 2:**
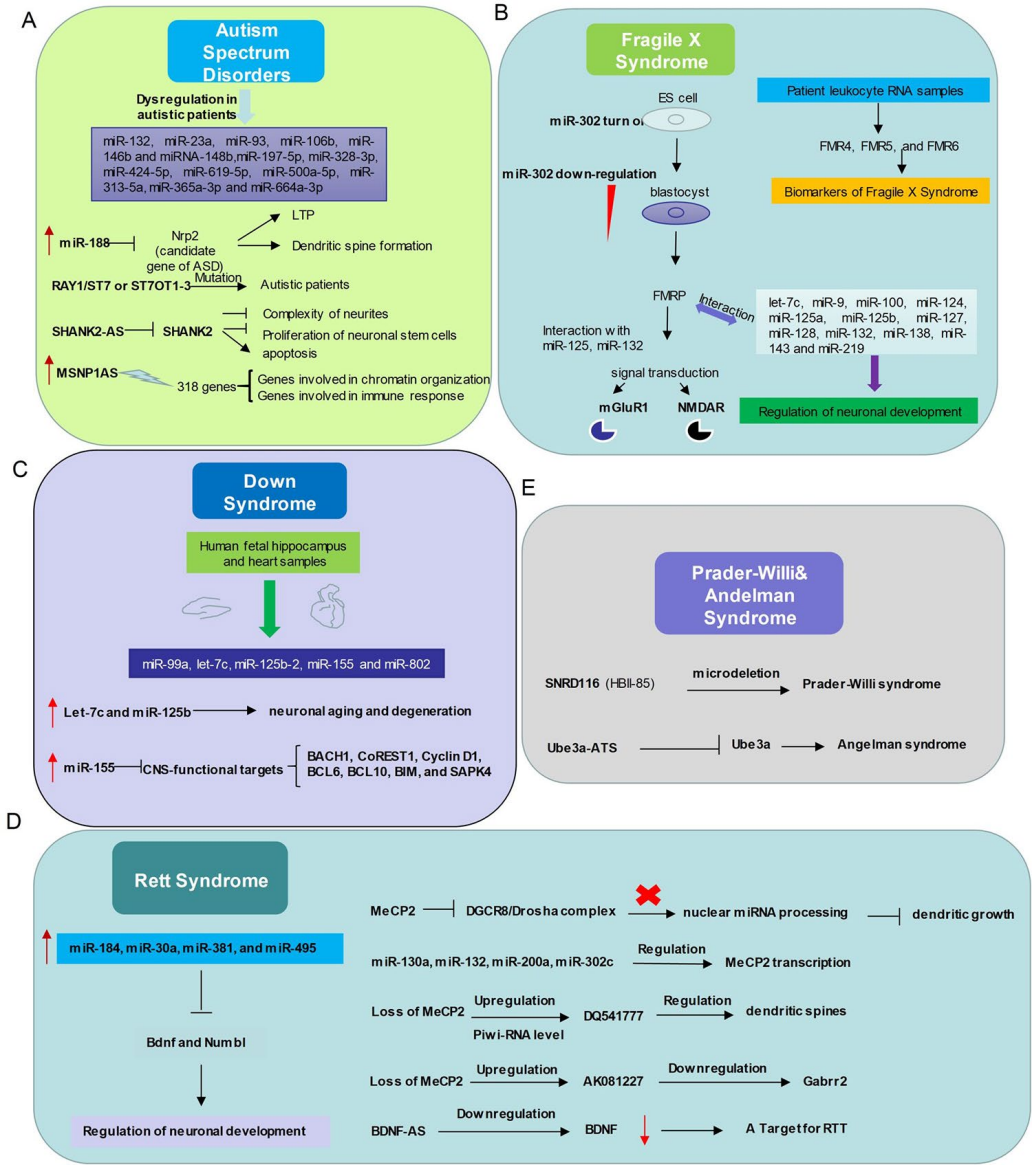
Relationship between non-coding RNAs and neurodevelopmental disorders. **(A)** In Autism Spectrum Disorders, miR-132, miR-23a, miR-93, miR-106b, miR-146b and miRNA-148b, miR-197-5p, miR-328-3p, miR-424-5p, miR-619-5p, miR-500a-5p, miR-313-5a, miR-365a-3p and miR-664a-3p expression levels are abnormal. miR-188, RAY1/ST7 or ST7OT1-3, SHANK2-AS, MSNP1AS participate in ASD with distinct ways. **(B)** The interactions between non-coding RNAs and mechanism diagram of Fragile X Syndrome. microRNAs and long non-coding RNAs regulate Fragile X Syndrome process and might be a class of biomarkers of Fragile X Syndrome. **(C)** Dysregulation of microRNAs related to Down Syndrome in human fetal hippocampus and heart samples. **(D)** Left, the expression level of several microRNAs up-regulates in Rett Syndrome patients and these microRNAs play important roles in modulating neuronal development. Right, relationship between Mecp2 and Non-coding RNAs. **(E)** Microdeletion of SnoRNA SNRD (HBI-85) leads to Prader-Willi syndrome like phenotype. Long non-coding RNA Ube3a-ATS represses Ube3a, which gives rise to Angelman syndrome.

Abnormal expression levels of miRNAs, including miR-132, miR-23a, miR-93, miR-106b, miR-146b and miRNA-148b, were observed in the serum, lymphoblastoid cells, or cerebellar cortex of autistic patients (Abu-Elneel et al., 2008; Talebizadeh et al., 2008; Tewarit Sarachana1 2010; Mundalil Vasu et al., 2014) (**Figure 2**). Many autism susceptibility genes are predictive targets of these differently expressed miRNAs, which further strengthens the causal relationship between miRNAs and autism (Abu-Elneel et al., 2008; Talebizadeh et al., 2008; Tewarit Sarachana1, 2010; Constantin, 2017).

A profiling study of circulating serum miRNAs in children with ASD reveals that differentially expressed miRNAs in serum may be involved in the molecular pathway of ASD (Kichukova et al., 2017), and serum miR-197-5p, miR-328-3p, miR-424-5p, miR-619-5p, miR-500a-5p, miR-313-5a, miR-365a-3p and miR-664a-3p may be served as potential biomarkers of ASD (Kichukova et al., 2017) (**Table 1**). Moreover, the interaction between miRNAs and autism risk genes in the cerebellum was observed in animal models of ASD. For example, miR-188 is down-regulated in autism patient (Trindade et al., 2013) and is up-regulated in response to long-term potentiation (Lee et al., 2012). MiR-188 promotes dendritic spine formation by blocking the expression of neuropilin-2, a well-known candidate gene of ASD (Lee et al., 2012) (**Figure 2**).

More than 200 lncRNAs are differentially expressed in the prefrontal cortex and cerebellum of ASD patients (Rennert, 2013). The autism loci containing RAY1/ST7 (suppression of tumorigenicity 7) encode at least four non-coding genes (ST7OT1-4, ST7AS1-4) which are located in the sense or antisense chains that potentially regulate RAY1/ST7. Several rare mutations of RAY1/ST7 or ST7OT1-3 genes have been detected in autistic patients (Vincent et al., 2002; van de et al., 2013) (**Figure 2**).

Genome-wide differential expression analysis of blood samples from ASD patients (Carninci et al.) identified 2407 up-regulated and 1522 down-regulated lncRNAs in peripheral blood leukocytes of ASD. The pathway enrichment analysis of these differently expressed lncRNAs revealed that they were mainly involved in synaptic vesicle circulation, long-term inhibition and long-term potentiation of neural pathways (van de et al., 2013). Differential expression of lncRNAs *SHANK2-AS* and *BDNF-AS* was also observed in ASD (van de et al., 2013; Wang et al., 2015; Tang et al., 2017) (**Figure 2**). In neurons, *SHANK2-AS* and *Shank2* can form double-stranded RNA that inhibit the expression of *Shank2*. Overexpression of *SHANK2-AS* reduces the complexity of neurites, and inhibits the proliferation of neuronal stem cells and promotes their apoptosis (Luo et al., 2018).

Moesin is a protein that in human is encoded by the MSN gene, which regulates neuronal structure and immune response. MSN-binding *MSNP1-AS* is highly expressed in postmortem cerebral cortex samples from individuals with ASD. Increased *MSNP1-AS* expression is also observed in individuals carrying the ASD-associated rs4307059 T allele (Kerin et al., 2012) (**Figure 2**). By targeting the *MSNP1-AS* gene promoter, *MSNP1-AS* knockdown disrupts the expression of 318 genes in neuroblastoma neural progenitor cells, many of which are involved in chromatin organization and immune response, indicating multiple transcriptional and translational functions of *MSNP1AS* in ASD-relevant biological processes (DeWitt et al., 2016) (**Table 1**).

### Fragile X Syndrome

FXS is the most common inherited cause of mental disorder and ASD (Lin, 2015; Hecht et al., 2017). FXS is caused by FMR1 (fragile X mental retardation 1) inactivation or dysfunction (Verkerk et al., 1991). FMR1 is required for normal neuronal connectivity and plasticity. The *FMR1* gene contains a CGG-repeat present in the 5′UTR which can be unstable upon transmission to the next generation. FXS patients have a repeat length exceeding 200 CGGs that generally leads to methylation of the repeat and the promoter region, resulting in silencing of *FMR1* gene expression (Fu et al., 1991; Dominique Heitz et al., 1992; Tang et al., 2017).

Several miRNAs have already been proven to involve in the development of FXS (Lin, 2015). During the embryonic stage, miR-302 is specifically expressed by embryonic stem cells, and it blocks the translation of FMR1, which is required to repress differentiation. At blastocyst stage, down-regulation of miR-302 promotes FMRP synthesis and subsequent neuronal development. In the normal neuronal development, FMRP, as an RNA-binding protein, interacts with miR-125 and miR-132 to regulate the signal transduction of metabolic glutamate receptors (mGluR1) and N-methyl-D-aspartate receptors (NMDAR) (Lin, 2015) (**Figure 2**). In addition, let-7c, miR-9, miR-100, miR-124, miR-125a, miR-125b, miR-127, miR-128, miR-132, miR-138, miR-143 and miR-219 might also interact with fragile X mental retardation protein (FMRP) to regulate neuronal development (Lin, 2015) (**Table 1**).

LncRNAs have recently emerged to influence the pathogenesis of FXS (Esteller, 2011; Taft et al., 2010; van de et al., 2013). The FMR1 bidirectional promoter is capable of translating lncRNA *FMR4* or *FMR1-AS1* which is an antisense transcription that overlaps the CGG repeat region (Khalila 2009; Ladd et al., 2007) (**Figure 2**). *FMR4* plays a critical role in regulating cell cycle, proliferation, and apoptosis of human neural precursor cells (Khalila 2009).

LncRNAs FMR5 and FMR6 have recently been linked to FXS (Pastori et al., 2014) (**Figure 2**). FMR5 is a sense lncRNA transcribed upstream of the FMR1 promoter, while FMR6 is an antisense transcript overlapping the 3′-UTR of FMR1. The expression of FMR4, FMR5, and FMR6 is detectable in the majority of patient leukocyte RNA samples, suggesting that it may be reliable biomarkers for FXS (Wahlestedt, 2013).

### Down Syndrome

DS is a neurodevelopmental disorder caused by the presence of all or part of a third copy of chromosome 21 (Asim et al., 2015). This disorder has been characterized with many clinical manifestations, including dementia, defects of the immunity system and congenital heart, and abnormalities of facial growth, gastrointestinal tract, and endocrine system (Malinge et al., 2009). Five miRNAs (miR-99a, let-7c, miR-125b-2, miR-155 and miR-802) have been found to be overexpressed in human fetal hippocampus and heart samples from individuals with DS (Fillat and Altafaj, 2012) (**Figure 2**). Let-7c and miR-125b have been shown to enhance neuronal aging and degeneration (Chawla et al., 2016). Recent evidence suggests that DS dementia strongly correlates with overexpression of miR-155 on chromosome 21 with concomitant reduction of multiple CNS-functional targets, including BACH1, CoREST1, Cyclin D1, BCL6, BCL10, BIM, and SAPK4 (Tili et al., 2018) (**Figure 2**).

### Rett Syndrome

RTT is a neurodevelopmental disorder caused by the loss of function of methyl-CpG-binding protein 2 (MeCP2) (Chahrour and Zoghbi, 2007; Obiols-Guardia and Guil, 2017). Because chromosome Y does not exist MeCP2, the disease occurs almost entirely in women (Weng et al., 2011; Obiols-Guardia and Guil, 2017). MeCP2 protein is highly expressed in neurons, acting as a transcriptional repressor and activator, depending on the context (Luikenhuis et al., 2004). Growing evidence suggests that various non-coding RNAs might play important roles in the development of RTT (Obiols-Guardia and Guil, 2017).

Due to direct or indirect deregulation following MeCP2 loss of function, disrupted miRNA expression has been reported in the disease progress of RTT (Urdinguio et al., 2010; Lyu et al., 2016). For instance, miR-184, miR-30a, miR-381, and miR-495 are aberrantly up-regulated in MeCP2 knockout mice (Nomura et al., 2008; Wu et al., 2010) (**Figure 2**). These miRNAs are known for repressing the expression of important modulators of neuronal development, such as *Bdnf* and *Numbl* (Liu et al., 2010; Wu et al., 2010). MeCP2 also interacts with pri-miRNA processing machines and affects their activity. For example, DGCR8/Drosha complex can be inhibited by MeCP2, thus affecting nuclear miRNA processing and dendritic growth (Cheng et al., 2014). Interestingly, miRNAs can also regulate MeCP2 transcription, such as miR-130a (Zhang et al., 2016), miR-132 (Lyu et al., 2016), miR-200a, and miR-302c (Rodrigues et al., 2016) (**Figure 2**). Future studies on the link between MeCP2 and the miRNA population will broaden our knowledge of regulatory network affected in RTT and will help develop better therapeutic strategies.

PiRNA expression levels are altered globally in the absence of MeCP2 (Saxena et al., 2012). There are at least 12 hippocampus-abundant piRNAs up-regulated with a fold change of over 1.5 in the cerebellum of MeCP2 KO mice. Among them, DQ541777, which is implicated in regulating the size of dendritic spines, is the 5th most abundant piRNA in the cerebellum libraries (Lee et al., 2011; Saxena et al., 2012) **Figure 2**. More specific functions of these dysregulated piRNAs in the pathophysiology of RTT are expected to be discovered in near future.

The aberrant lncRNA transcriptome is also present in the brain of RTT mice. For instance, the AK081227 and AK087060 transcripts are up-regulated in MeCP2-null brains (Petazzi et al., 2013). The overexpression of AK081227 mediated by the Mecp2 loss is associated with the down-regulation of its host coding protein gene *Gabrr2*, a major inhibitory neurotransmitter in the mammalian brain where it acts at GABA receptors, which are ligand-gated chloride channels (Petazzi et al., 2013). The neurotrophic BDNF is known to be aberrantly diminished in RTT individuals (Katz et al., 2016), it can be speculated that the lncRNA BDNF-AS might be an important therapeutic target for treating RTT. Although lncRNAs play an important role in neuronal development, their roles in the pathogenesis of RTT is largely unknown (Mo 2015; Lin et al., 2014; Ng et al., 2013).

### Prader-Willi Syndrome and Angelman Syndrome

Chromosome 15q11-q13 is a region containing a lot of genomic imprinting genes (Kalsner and Chamberlain, 2015). Prader-Willi Syndrome (PWS) and Angelman Syndrome (AS) are two different types of neurodevelopmental disorders which are caused by loss of function or overexpression of at least one imprinted gene at the 15q11-q13 locus (Kalsner and Chamberlain, 2015). PWS is characterized by intellectual disability, irritability, short stature, and low fertility/hypogonadism (Buiting, 2010). NECDIN and small ribonucleoprotein polypeptide N (SNRPN) are functionally related to the pathological features of the disease (Francoise Musctelli, 2000). SNRPN downstream introns contain SNRD116 (HBII-85) snoRNA clusters, and paternal genetic microdeletion of SNRPN clusters might lead to PWS (Sahoo et al., 2008; de Smith et al., 2009) (**Figure 2**). Actually, mice with deletion of MBII85 snoRNA clusters demonstrated obvious PWS phenotypes (Skryabin et al., 2007), indicating non-coding RNAs can be tightly regulated and may play critical roles in the pathology of PWS.

The pathological features of AS include delayed development, intellectual disability, severe speech impairment, and problems with movement and balance (Buiting, 2010). AS is caused by the deletion and/or mutation of *Ube3a* on the maternal chromosome. While in patients with AS the maternal *Ube3a* allele is inactive, the paternal allele is intact but epigenetically silenced through the *Ube3a-ATS* part of LNCAT (large non-coding antisense transcript) at the Ube3a locus (Runte et al., 2001). Elucidating the mechanisms of how Ube3a-ATS involves in silencing the paternal *Ube3a* may lead to new therapies for AS (Landers et al., 2005; Meng et al., 2012) (**Figure 2**).

### Conclusions

Non-coding RNAs have emerged as important regulators in the brain development and function. Although the number and functional subclasses of non-coding RNAs has steadily increased, it still likely represents only a small fraction of the total RNA transcriptome underlying the ontogeny and functional complexity of mammalian brain functions in health and disease. Aberrant expression of non-coding RNAs has linked with various neurodevelopmental diseases. The regulatory network of non-coding RNAs in neurodevelopmental disorders is very complicated, and the molecular mechanisms of non-coding RNA causing neurodevelopmental diseases are still largely unknown. However, we still hope that this review raises awareness of the central roles that large non-coding RNAs and their complex regulatory networks in brain development and function.

With more defined molecular function and mechanism, Non-coding RNAs have the great potential to serve as disease biomarkers or drug targets, especially in neurodevelopmental disorders. For example, miR-132, miR-23a, miR-93, miR-106b, miR-146b and miRNA-148b are abnormally expressed in autistic patients (Mahesh Mundalil Vasu1, 2014; Trindade et al., 2013). miR-99a, let-7c, miR-125b-2, miR-155 and miR-802 are found to be overexpressed in human samples from individuals with Down Syndrome(Fillat and Altafaj, 2012). In fragile X syndrome, the expression of FMR4, FMR5, and FMR6 is detectable in the majority of patient leukocyte RNA samples, suggesting that it may be reliable biomarkers for Fragile X Syndrome (Wahlestedt, 2013). The analysis and integration of this information with other datasets will get new clues of non-coding RNAs as reliable biomarkers.

A large number of studies have suggested that non-coding RNAs might be new promising targets for the treatment of neurodevelopmental disorders. However, several challenges remain to be investigated before non-coding RNAs can be routinely used in outbreak investigation and clinical practice. Firstly, more efficient non-coding RNAs delivery systems are needed to develop. Secondly, the biology of non-coding RNAs, such as their structural motifs, stability, degradation, and gene regulatory network, needs further investigations. Finally, preclinical research and clinical trials are required to determine the safe dose and therapeutic potentials of non-coding RNAs.

In addition, how non-coding RNAs operate in CNS at the molecular, cellular and more hierarchical neural network levels still remains elusive. Therefore, it is important to discover molecules based on further elucidating more pathways of non-coding RNA roles in the CNS and how non-coding RNA dysfunction leading to neurodevelopmental disorders. Based on more understanding of non-coding RNAs in the CNS, the researchers will probably develop new diagnostic and therapeutic approaches for neurodevelopmental disorders.

## Author Contributions

C-ML directed the manuscript preparation. S-FZ, JG, and C-ML wrote the manuscript.

## Conflict of Interest

The authors declare that the research was conducted in the absence of any commercial or financial relationships that could be construed as a potential conflict of interest.
